# Sulphotransferase-mediated toxification of chemicals in mouse models: effect of knockout or humanisation of SULT genes

**DOI:** 10.1042/EBC20240030

**Published:** 2024-12-04

**Authors:** Hansruedi Glatt, Walter Meinl

**Affiliations:** 1 Federal Institute for Risk Assessment (BfR), Department Food Safety, Max-Dohrn-Strasse 8-10, 10589 Berlin, Germany; 2 German Institute of Human Nutrition (DIfE) Potsdam-Rehbrücke, Department of Nutritional Toxicology (HG & WM) and Department of Molecular Toxicology (WM), Arthur-Scheunert-Allee 114-116, 14558 Nuthetal, Germany

**Keywords:** DNA adducts, model organisms, sulphotransferases, SULT1A1, toxification

## Abstract

Cytosolic sulphotransferase (SULT) enzymes catalyse reactions involved in xenobiotic elimination and hormone regulation. However, SULTs can also generate electrophilic reactive intermediates from certain substrates, including the activation of carcinogens. Here, we review toxicological studies of mouse strains with SULT status altered by genetic modification. Knockout mouse strains have been constructed for the enzymes Sult1a1, 1d1, 1e1, 2b1 and 4a1. In addition, transgenic strains are available for human SULT1A1/2. Among SULT knockout mouse strains, reduced fertility (Sult1e1) and early postnatal death (Sult4a1) were observed. In contrast, Sult1a1 or Sult1d1 knockouts and SULT1A1/2 transgenics were healthy and showed no obvious deficiencies. These strains were used in toxicological studies with 13 chemicals. Manipulation of the SULT system altered dramatically the adverse effects of many compounds; thus, very large differences in levels of DNA adducts formed in the liver or other tissues were seen with some chemicals – up to 99.2% decreases in knockouts and 83-fold increases in SULT1A1/2 transgenics. In many cases, these changes were restricted to the tissues in which the corresponding enzymes are expressed, arguing for local activation. However, with some compounds, the kidney was an important target tissue, due to the active transfer to that organ, via the circulation, of reactive sulphuric acid esters.

## Introduction

### Sulphotransferase-mediated toxification reactions

Cytosolic sulphotransferases (SULTs) play important roles in xenobiotic biotransformation and in the regulation of several hormones. The enzymes transfer SO_3_^−^ (variously referred to as the ‘sulpho’ or ‘sulphonate’ group) from the donor substrate 3'-phosphoadenosine-5'-phosphosulphate (PAPS) to a nucleophilic atom (O, N, S) of their acceptor substrates; C-sulphonation was observed for α,β-unsaturated carbonyl groups [1].

The most common reaction, sulpho transfer to an oxygen atom (usually a hydroxyl group), results in the formation of a sulphuric acid ester. These esters are fully dissociated under physiological conditions, *i.e.*, they exist as sulphates. The reaction can be termed either sulphation (with respect to the product formed) or sulphonation (with respect to the transferred group).

The anionic character of the sulphate group facilitates product excretion in two ways: Firstly, water solubility is greatly increased; for example, the 1-octanol/water partition coefficient of the hydrophobic hormone 17β-oestradiol (*P* = 490) is decreased to <0.01 by 3-sulphation. Secondly, since permanent ions cannot penetrate cell membranes passively, directional transport, supported by transmembrane transporters, can result in excretion of the conjugates into urine, bile and/or gut. For these reasons conjugation reactions with anionic groups (such as sulphonation or glucuronidation) are often associated with detoxification.

**Scheme 1:**
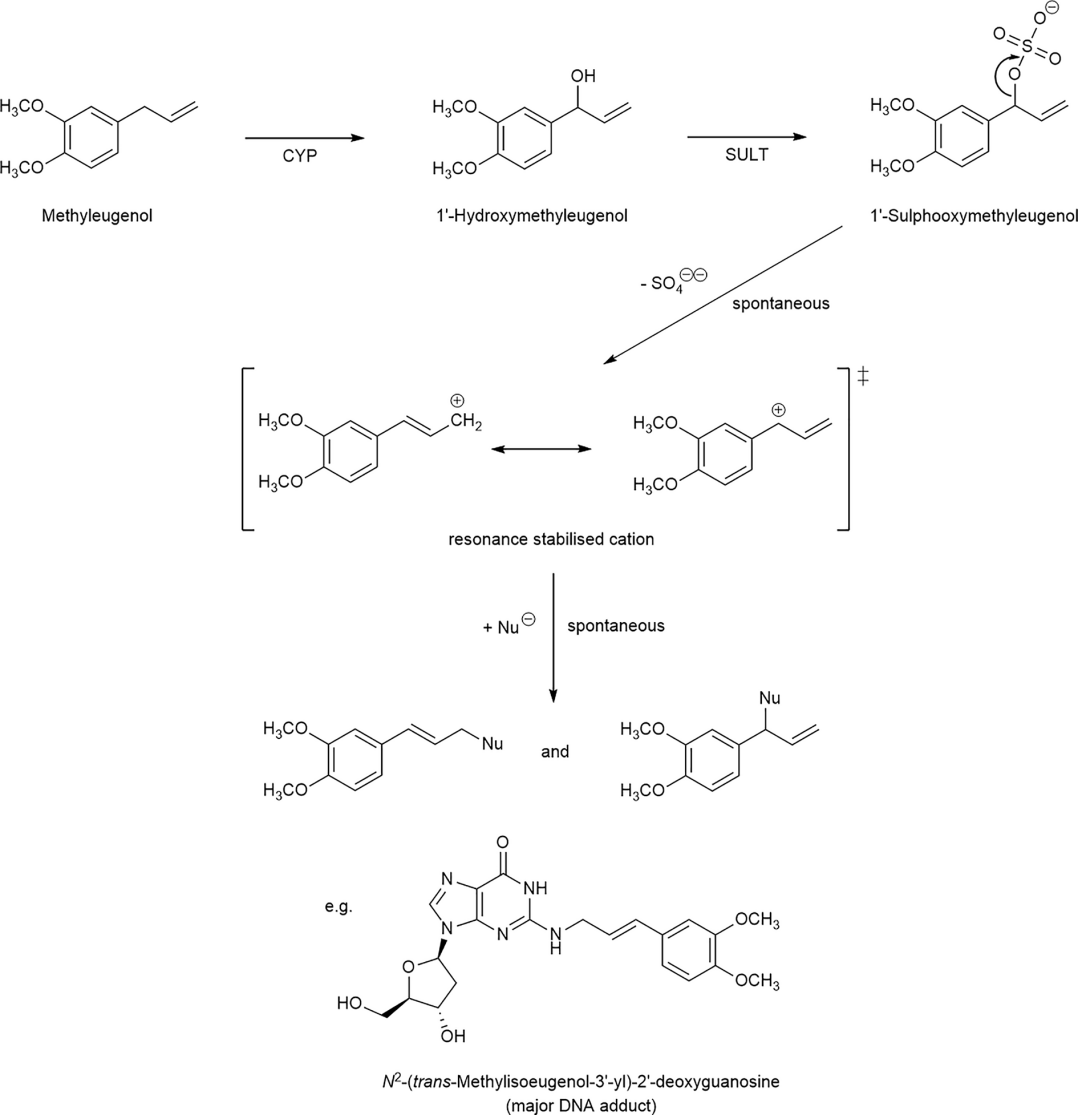
Bioactivation of methyleugenol to an electrophilic sulphuric acid ester. CYP, cytochrome P450; SULT, sulphotransferase; Nu^–^ (or NuH - H^+^), nucleophile (*e.g.*, water, glutathione, or nitrogen atoms in proteins and nucleic acids).

Nevertheless, activation processes can also result. Sulphuric acid is a strong acid (p*K*_a_ = 1.9 for dissociation of the second proton). Thus, the bonds between the sulphate group and the H atoms readily undergo heterolytic cleavage, releasing H^+^. Analogous heterolytic cleavage may occur with some metabolically formed sulphuric acid esters (Scheme 1). In such cases, carbonium and/or nitrenium ions, rather than H^+^, are released; the products are strong electrophiles that can covalently bind to nucleic acids, proteins, or other biomolecules. Heterolytic cleavage is favoured if the resulting cation is stabilised by inductive effects (electron-donating groups) or by resonance (in aromatic and other conjugated systems). SULT-mediated toxification leading to chemically reactive intermediates has been observed with the following classes of compounds:

- Hydroxylamines formed from aromatic amines and nitroarenes: Many homo- and heterocyclic aromatic amines (*e.g*. 4-aminobiphenyl [2] and 4-aminoazobenzene [3]) and nitroarenes (*e.g*. 3-nitrobenzanthrone [4] and aristolochic acids [4]) are established carcinogens in animal models or humans. Typically, these toxicants are metabolically activated via formation of hydroxylamines (‘proximate’ carcinogens) to reactive esters, most often to sulphates (by SULTs) and acetates (by N,O-acetyltransferases, NATs). In line with the much stronger acidity of sulphuric acid compared with acetic acid, the sulphates are more reactive and shorter lived than the corresponding acetates.
- Aromatic hydroxylamides: *N*-Sulphooxy-2-acetylaminofluorene was the first electrophilic metabolite of a carcinogen to be discovered [5].
- Benzylic alcohols: These compounds are common metabolites of alkylated polycyclic aromatic hydrocarbons, industrial chemicals, pharmaceutical drugs, and some plant secondary metabolites, *e.g*. 1-methylpyrene [6], furfuryl alcohol [7,8], nevirapine [9], hycanthone [10], and methyleugenol [11]. The structural formulas and activation pathways of 1-methylpyrene and methyleugenol are shown in Schemes S1 and 1, respectively.
- Propenylic alcohols attached to aromatic structures: In this case, the resonance stabilisation involves an allylic bond that is further conjugated with an aromatic system. Examples are α-hydroxytamoxifen [12] and 3’-hydroxymethylisoeugenol [13].
- Allylic alcohols such as α- and β-hydroxycyproterone acetate [14].
- Secondary nitroalkanes: 2-Nitropropane, an industrial chemical, induces hepatocellular carcinomas in animal models. In water, it isomerises to propane-2-nitronate. The compound forms aminated and oxidised bases in the DNA and induces mutations in cells expressing SULTs [15,16]. Some other secondary nitroalkanes were activated in a SULT-dependent manner, but the SULT isoforms involved were not identified [17].

Literature searches (including [2–16,18–25]) have identified 105 compounds that can be converted into reactive, genotoxic, or cytotoxic intermediates in cell models engineered for the expression of specific SULT isoforms or by purified SULT enzymes. In addition, we have unpublished data demonstrating toxification of about 20 other chemicals.

These findings may only represent the ‘tip of the iceberg’. Lificiguat (YC-1), an activator of soluble guanylyl cyclase, is highly active against a large number of cell lines derived from cholangiocarcinomas and hepatocellular carcinomas. SULT1A1 expression was important for this activity, rendering YC-1 a strong electrophile that covalently binds to proteins [24]. Likewise, a large number of congeners (>140) of YC-1 (containing furfuryl alcohol in their structure) and AHBAs (sharing a core structure of 4-Amino-2-HaloBenzyl Alcohol) were much more cytotoxic in two cancer cell lines with high SULT1A1 expression than in two similar SULT1A1-deficient lines [24]. The authors of this study conducted computational analyses on compounds that had been tested for cytotoxicity in many cancer cell lines; they identified hundreds of compounds whose activity in different cell lines showed high correlation with either SULT1A1 transcript levels or YC-1 sensitivity. Structural elements suitable for SULT-mediated activation were present in all of these compounds (*e.g*. furfuryl alcohol, benzyl alcohol, *N*-substituted indole-3-carbinol, or an amino group on an aromatic ring).

In summary, much knowledge has been obtained regarding the activation of chemicals by individual SULT isoforms *in vitro* and these findings need to be verified *in vivo*, in appropriate animal models and in humans.

## The SULT superfamily in the human and the mouse

Based on both their sequences and their structures, the SULTs are seen to form a gene/protein superfamily, comprising 13 functional genes in humans [26,27] and 21 in mice [28]. Interestingly, the distribution of the genes differs markedly. Subfamilies 1D, 3A, 5A, and 7A are present in functional form in mice but not in humans. In contrast, the SULT1A subfamily comprises only a single functional gene (*SULT1A1*) in mice (and all other non-primate species studied), whereas humans have four functional *SULT1A* genes [26]. (Note: Following standard convention, we use upper case for human genes (italics) and enzymes (romans), *e.g*., SULT1A1, and lower case with first-letter capitalization for isoforms of the mouse, *e.g*. Sult1a1. Upper case is also used for generic designations.)

Mouse strains with manipulated *SULT1A* and *SULT1D* status have been used for toxicological studies and this discussion will focus on these particular enzyme subfamilies. The amino acid sequences of the human and murine isoforms are shown (Figure S1).

SULT1A1 is the most abundant SULT isoform in adult human liver, at the protein level [29], and it is also present in many other tissues, including gut, kidney, lung, prostate, brain, and blood platelets [29–32]. The enzyme displays unusually broad substrate tolerance, ranging from very small molecules (*e.g*., ethanol [33], minoxidil (a cation) [34], and propane-2-nitronate (an anion) [15,16]) to large molecules such as 6-hydroxymethylanthanthrene and 2-hydroxy-3-methylcholanthrene [35]. Accordingly, far more toxification reactions have been characterised with this isoform (85 compounds studied with recombinant cells or purified enzymes) than with any other. SULT1A2 differs only in eleven amino acid residues (out of 295) from SULT1A1, as illustrated in Figure S1 (these numbers refer to the major allelic variants).

SULT1A2 protein has been detected in human liver and caecum samples, but at clearly lower levels than those of SULT1A1 [30]. The majority of substrates is more efficiently metabolised by SULT1A1, compared with SULT1A2, at similar protein levels. Conversely, a small number of substrates, including *N*-hydroxy-2-acetylaminofluorene [5] and ethanol [33], is preferentially conjugated by SULT1A2. Hesperetin shows substrate inhibition by SULT1A1 even at very low concentrations, whereas it is conjugated efficiently by SULT1A2 over a wide range of substrate concentrations [36].

The *SULT1A3* and *SULT1A4* genes (common allelic variants) encode an identical protein sequence (usually termed SULT1A3), differing in 20 amino acid residues from SULT1A1 (Figure S1). SULT1A3 is characterised by high catalytic efficiency toward L-dopamine and other catecholamines and a narrower substrate tolerance toward xenobiotics, compared with SULT1A1 [37,38] and SULT1A2. SULT1A3 is practically absent from adult liver, but particularly high in small and large intestine; brain, lung, kidney and blood platelets are other expression sites [29–31].

Evolution of the SULT1A genes has been accompanied by functional diversification, regarding both substrate specificity and regulation (including tissue distribution) of the paralogous enzymes in primates. Mouse Sult1a1 shares many substrates with human SULT1A1 [38]. However, this is not true for all substrates – for example, human SULT1A1 was 7- to 20-fold more active toward 4-hydroxy-2-amino-1-methyl-6-phenylimidazo[4,5-*b*]pyridine (4-hydroxy-PhIP) than its orthologues from the mouse, rat, or rabbit [38]. Likewise, the secondary benzylic alcohol 2-hydroxy-3-methylcholanthrene was specifically activated by human SULT1A1 expressed in *Salmonella typhimurium*, but not by its orthologues from mouse, rat, or dog [35]. Significant levels of mouse Sult1a1 protein were detected only in the liver and large intestine (appreciable mRNA, but not protein, expression was found in the lungs) [39,40].

Among the mouse Sult isoforms, Sult1d1 is most similar to Sult1a1, regarding amino acid sequences [1] (for amino acid sequences see Figure S2). Sult1d1 efficiently catalyses the conjugation of small phenols (as for SULT1A1 enzymes) and catecholamines (as for human SULT1A3); like human SULT1A1, it activates some promutagens more efficiently than does mouse Sult1a1 – examples are *N*-hydroxy-PhIP, 5-hydroxymethylfurfural (HMF), and furfuryl alcohol [41]. Mouse Sult1d1 is expressed in many tissues, with particularly high levels in small and large intestine and kidney [39,40]. Looking at these overlapping characteristics, the expansion of the SULT1A subfamily in primates may have rendered SULT1D1 superfluous (in humans, it is present only as a pseudogene [42]).

## Mouse models with SULT status altered by genetic technology

Several mouse strains with modified SULT status were constructed in our laboratory; among these, the following strains were used for toxicological studies considered in the present review:

- A genomic sequence comprising the transcribed and long flanking regions of *SULT1A1* and *SULT1A2* was introduced into oocytes of FVB/N mice [43]. Several strains carrying the human transgene were obtained, they differed in the transgene copy numbers and integration sites. All toxicology studies were conducted in the strain termed ‘tg1’ [43], which carries multiple copies of the human gene cluster at a single integration site on chromosome 9. SULT1A1 protein expression was detected in 14 of 16 tissues studied; SULT1A2 protein was found in 11 tissues, at markedly lower levels (∼1/4 on the average) than SULT1A1.
- Sult1a1-knockout (ko) mice were produced from successfully recombined embryonic stem cell clones, which were injected into blastocysts [44]. Some of the resulting chimeric mice carried the Sult1a1-ko in germ cells, allowing the generation of mice with Sult1a1-ko in all cells. These mice were bred into the FVB/N background [44].
- Sult1d1^−/−^ mice were constructed using the same procedures as for the Sult1a1^−/−^ strain (using a recombined stem cell clone) [45].
- By appropriate breeding, the following strains were obtained (all strains were homozygous with regard to the SULT loci indicated):
  – Sult1a1^−/−^
  – Sult1d1^−/−^
  – Sult1a1^−/−^ Sult1d1^−/−^
  – SULT1A1/2^+/+^ [with intact wild-type (wt) *Sult1a1* and *Sult1d1* genes]
  – SULT1A1/2^+/+^-Sult1a1^−/−^
  – SULT1A1/2^+/+^-Sult1a1^−/−^-Sult1d1^−/−^
  The strains Sult1a1^−/−^, Sult1d1^−/−^ and Sult1a1^−/−^-Sult1d1^−/−^ were used directly for experiments. Strains carrying the human transgene were bred with wt (or appropriate ko mice) to obtain F1 mice hemizygous for the transgene (but homozygous for the ko indicated). Unless specified otherwise, these F1 mice were used for the experiments.

Other research groups constructed the following mouse strains:

- A Sult1a1^−/−^ mouse strain was used to study a possible role of indoxyl sulphate, a uremic toxicant, in the progression of renal fibrosis and acute cisplatin-induced nephrotoxicity [46,47]. This strain was generated in the C57BL/6J background, whereas our Sult1a1^−/−^ mouse is in the FVB/N background.
- Sult1e1^−/−^ mice [48].
- The same group has generated a mouse strain expressing Sult1e1 via the aP2 (adipocyte protein 2) promoter; this manipulation led to selective overexpression of Sult1e1 in macrophages and adipocytes [49].
- Sult2b1^−/−^ mice are available from the Jackson Laboratory (Bar Harbor, ME, USA; strain 018773) [50].
- Mice with Sult2b1 deletion specifically in intestinal epithelial cells (Sult2b1^f/f^ Villin-Cre mice) [51]
- Sult4a1^−/−^ mice (2 strains, differing in the deletions leading to gene disruption) [52].

## The effects of genetic manipulation of SULTs on the vitality, fertility and other physiological parameters of mice

SULTs are important regulators of numerous hormones (and hormone precursors), including dehydroepiandrosterone (DHEA) [53], testosterone [53], pregnenolone [54], 6β-oestradiol [55], 6β-oestrone [55], iodothyronines [56,57], dopamine [58] and other catecholamines [38], serotonin [38], vitamin D3 [59], and 6-hydroxymelatonin [38]. Often the sulphates serve as storage forms, the back-reaction being catalysed by sulphatases [56,60]. Furthermore, some sulphates of steroids and other hormones have direct physiological activities different from those of the unconjugated hormones [61–64]. Bile acids [53], cholesterol [50,51,54], as well as 6-hydroxydopamine and 7-hydroxyserotonin (toxic compounds formed from monoamines by autoxidation) [65] are further endogenous substrates of SULTs. Due to these numerous endogenous functions of SULTs, their genetic manipulation might impair some physiological processes. Indeed, Sult1e1^−/−^ mice produce fewer and smaller litters than wt mice [66], due to age-dependent damage in the testis [66] and placental thrombosis [48]. Sult4a1^−/−^ pups show progressive neurological disorders and die around postnatal days 21–25 [52]. SULT1E1/Sult1e1 is the only isoform able to metabolise efficiently 6β-oestrone and 6β-oestradiol at low (physiological) concentrations (*K*_m_ ∼ 5 nM) [55]. Expression of SULT4A1 appears to occur only in the brain [52,67]; no SULT activity has been detected in all studies conducted, except one (using a peculiar substrate, 6-hydroxy-4-methylbenzo[*d*]thiazole-2-carbonitrile with mouse Sult14a1 expressed in fission yeast [27]); several researchers postulated that SULT4A1 has adopted new functions other than sulpho conjugation [67–69].

In contrast, our strains with Sult1a1-ko, Sult1d1-ko, and tg-SULT1A1/2 (in any combinations) showed normal fertility, weight development and behaviour. Furthermore, the global hepatic gene expression was compared in wt, SULT1A1/2^+/+^ and Sult1a1^−/−^ mice using arrays covering 39,000 transcripts. The genetic manipulations did not lead to altered expression of any genes encoding other SULT isoforms or other xenobiotic-metabolising enzymes. Among the remaining genes, only a handful genes showed changes in expression exceeding a factor of 1.5 in tg or ko mice compared with wt mice.

Likewise, Sult2b1^−/−^ mice are healthy and without any obvious deficiencies (www.jax.org/strain/018773 and [50]). However, they lack cholesterol sulphate in the dermis and intestinal cells (modifying immune reactions) [50].

## The effects of genetic manipulation of SULT1A1 on formation of DNA adducts by genotoxicants in mouse liver *in vivo*

**Figure 1:**
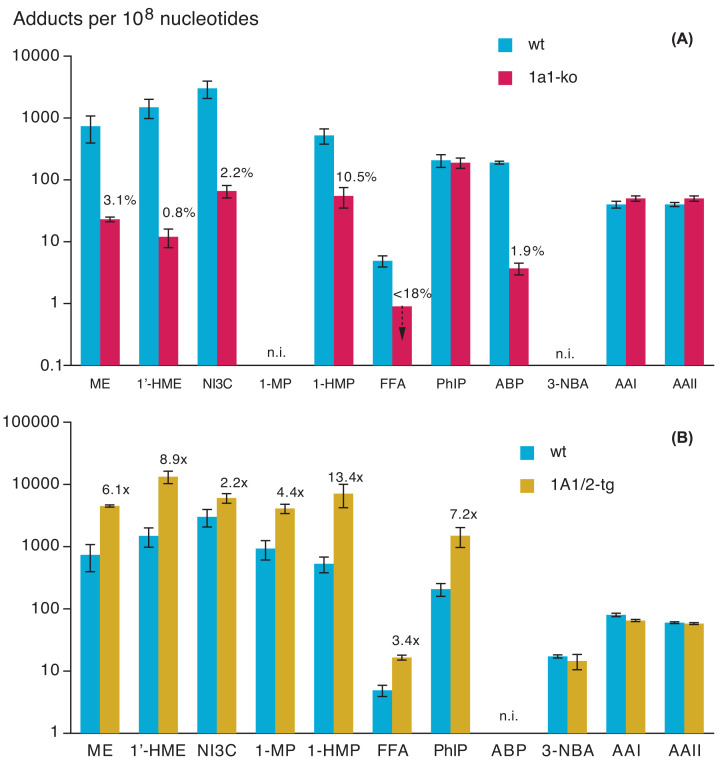
Sult1a1-knockout (upper panel) and transgenic human SULT1A1/2 (lower panel) effects on formation of hepatic DNA adducts in male mice Mice were treated with a single oral or i.p. dose of the indicated test compound. Values are means +/− SD of three to nine male mice (8–10 weeks old). The percent decreases or fold increases in adduct levels (compared with wt animals) are indicated, if the difference is statistically significant (*p* < 0.05, mostly much lower). If no numbers for decreases/increases are given, adduct levels do not differ with statistical significance from those in wt mice. Hemizygous transgenic mice were used, in the *Sult1a1-Sult1d1*-ko (FFA) or *Sult1a1-Sult1d1*-wt background (all other compounds). Adduct levels were determined using ^32^P-postlabelling (3-NBA, AAI, AAII) or isotope dilution LC-MS/MS (remaining compounds). Since the recovery of the adducts was unknown with the ^32^P-postlabelling method, values of adduct levels are only relative (RAL). When LC-MS/MS was used, data refer to a specific dG adduct (the chemical structures of two adducts are shown in Scheme 1 and S1; with various compounds, dA adducts were determined additionally; they were lower than, but paralleled, the dG adducts). n.i., not investigated; dashed arrow: no adducts were detected – the bar indicates the LOD. Methyleugenol (ME): 50 mg (280 µmol)/kg (p.o.) 6 h before sacrifice [44]; 1’-Hydroxymethyleugenol (1’-HME): 54.5 mg (280 µmol)/kg (i.p.) 6 h before sacrifice [11]; *N*-Methoxy-indole-3-carbinol (NI3C): 106 mg (600 µmol)/kg (p.o.) 8 h before sacrifice [40]; 1-Methylpyrene (1-MP): 108 mg (500 µmol)/kg (i.p.) 2 h before sacrifice [6]; 1-Hydroxymethylpyrene (1-HMP): 19.3 mg (83 µmol)/kg (i.p.) 30 min before sacrifice [45]; Furfuryl alcohol (FFA): 400 mg (4077 µmol)/kg (i.p.) 60 min before sacrifice [7]; 2-Amino-1-methyl-6-phenylimidazo[4,5-*b*]pyridine (PhIP): 90 mg (400 µmol)/kg (p.o.) 8 h before sacrifice [43] (updated by a new study using the same treatment scheme, but LC-MS/MS for adduct detection); 4-Aminobiphenyl (ABP): 20 mg (118 µmol)/kg (i.p.) 24 h before sacrifice [2]; 3-Nitrobenzanthrone (3-NBA): 2 mg (7.3 µmol)/kg (i.p.) 24 h before sacrifice [4]; Aristolochic acid I (AAI): 50 mg (146 µmol)/kg (p.o.) 24 h before sacrifice [4]; Aristolochic acid II (AAII): 50 mg (165 µmol)/kg (p.o.) 24 h before sacrifice [4].

The impact of Sult1a1-ko on the formation of hepatic DNA adducts was investigated with nine compounds. All of these compounds are toxified by human SULT1A1 in some *in vitro* models (comprehensive references for the *in vitro* data are given in the original publications on the adduct formation in the genetically modified mouse models [2,4,6,7,11,40,43,45]). Results for male mice are shown (Figure 1A). Sult1a1-ko dramatically diminished hepatic DNA adduction by six compounds, to below the limit of detection (LOD) with furfuryl alcohol or to 0.8–10.5% of the level in wt mice, implying that Sult1a1 was the principal activator. Four of these compounds are either known hepatocarcinogens (methyleugenol, 4-aminobiphenyl) or metabolites (proximate genotoxicants) of hepatocarcinogens [(1’-hydroxymethyleugenol, 1-hydroxymethylpyrene (1-HMP)] in mice. The other compounds displaying strong effects of Sult1a1 were furfuryl alcohol and *N*-methoxy-indole-3-carbinole (NI3C). Furfuryl alcohol induces kidney tumours in male mice [70]. NI3C (a decomposition product of 1-methoxy-3-indolylmethyl-glucosinolate present in cruciferous plants), which led to particularly high adduct levels in wt mice [compared with the other test compounds (Figure 1, blue bars)], has not yet been investigated regarding carcinogenicity.

Transgenic (tg) human SULT1A1/2 enhanced the hepatic DNA adduction by seven out of ten genotoxicants tested (Figure 1B). This finding demonstrates that human SULT1A1/2 efficiently activates the corresponding compounds, not only *in vitro*, but also in an *in vivo* setting (*i.e.*, at toxicologically relevant levels of the proximate genotoxicant, and in the presence of competing metabolic pathways).

In the liver, the impact of tg SULT1A1/2 was usually inverse to that of Sult1a1-ko; and it enhanced the adduct formation even in the presence of the endogenous Sult1a1 (in mice carrying the transgene in the Sult1a1 wt-background). Thus, the endogenous level of Sult1a1 was insufficient for maximal activation, presumably due to competition by other metabolic pathways for the proximate genotoxicant. Increases in adduction in SULT1A1/2-tg mice were strong, up to 13.4-fold (compared with wt mice). This might be due to higher catalytic efficiency of the human orthologue and/or the high expression of tg SULT1A1/2 compared with the endogenous Sult1a1.

Adduction by PhIP had unique characteristics. It was strongly enhanced by tg SULT1A1/2, but unaffected by Sult1a1-ko; this pattern was also observed in all extrahepatic tissues studied. This finding suggests differences in the substrate specificity between SULT1A1/2 and Sult1a1.

## The effects of genetic manipulation of SULT1A1 on DNA adduct formation in extrahepatic tissues

**Figure 2:**
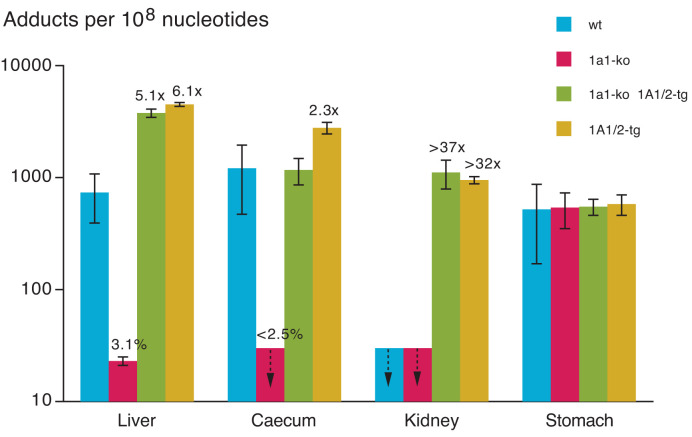
Sult1a1-knockout and transgenic human SULT1A1/2 effects on formation of DNA adducts by methyleugenol in four mouse tissues Data are from [44]. Male mice were treated with a single oral dose of methyleugenol (50 mg/kg body weight) 6 h before sacrifice. Values are means +/− SD of four mice (8–10 weeks old). Adduct levels refer to *N*^2^-(*trans*-methylisoeugenol-3′-yl)-2′-deoxyguanosine. Further details are as stated in the legend to Figure 1.

All genotoxicants described in Figure 1 were also studied with regard to formation of DNA adducts in extrahepatic tissues, nearly always giving positive results, at least in SULT1A1/2-tg mice. However, the influence of SULT1A1 manipulation on this adduction varied greatly among tissues, as exemplified by methyleugenol (Figure 2). Sult1a1-ko drastically decreased adduction in the liver and caecum, tissues with high Sult1a1 expression [39], but did not affect adduct formation in kidneys and stomach, where Sult1a1 mRNA levels were close to, or below, the LOD [39]. However, the latter tissues differed by the levels of adducts: < LOD in kidneys, high in stomach. Several Sult isoforms, such as Sult1b1, 1c1, 1c2, 1d1, 2b1, 3a1, and 5a1, are expressed in stomach [39] and might mediate the activation of methyleugenol. DNA adduction in the stomach is of particular interest, as methyleugenol induced various pathological changes in this organ, including some malignant tumours, in a long-term study in mice, although liver was the primary target of carcinogenesis [71].

SULT1A/2 enhanced the formation of methyleugenol DNA adducts in the liver, caecum and kidneys, sites of high SULT1A/2 protein expression in tg mice [43], but was ineffective in the stomach, a site showing only very low levels of SULT1A1 proteins in tg mice [43] and in humans [30].

The observation that the effects of SULT1A1 manipulation varied so strongly among tissues argues for local activation rather than transfer of the reactive intermediates between tissues. In particular, no methyleugenol-derived DNA adducts were detected in the kidney of wt mice despite extensive activation in the liver (and in other tissues). From this finding, we conclude that the high levels of renal adducts detected in SULT1A1/2-tg mice must be due to local activation of methyleugenol by SULT1A1/2.

Similar considerations apply to the other compounds and target tissues studied, as follows:

- Where Sult1a1-ko affected DNA adduct formation, the effect was seen mainly in the liver and large intestine, the major sites of Sult1a1 expression; with some compounds, also in kidney (much weaker effects than in the liver), as considered in the following paragraph; effects of Sult1a1-ko were absent or very weak in other extrahepatic tissues (with low or unknown Sult1a1 expression).
- In contrast, tg SULT1A1/2 enhanced adduction in many tissues, in accordance with the wide expression of SULT1A1/2 in tissues of tg mice [43]. Compared with adduction in wt mice, the effect (fold increase) was often particularly strong in the small intestine, kidney, and lung – tissues with high expression of SULT1A1/2 but low (or undetectable) levels of Sult1a1.
- Sporadically high levels of adducts were formed in an individual tissue with negligible influence of the SULT1A status, despite strong impact in liver. This was the case for the stomachs of mice treated with methyleugenol or NI3C. Another case in point is 4-aminobiphenyl, which is a urinary bladder carcinogen in humans. In mice, the compound induces tumours in the bladder (males > females) and liver (females > males), associated with the formation of high levels of DNA adducts. Sult1a1-ko practically abolished adduction in the liver [2] (and Figure 1A), but had much milder effects in the bladder – interestingly, these effects were bidirectional (depending on sex and treatment scheme) and closed the gender gap (in particular in Sult1a1-Sult1d1 double ko mice) [2]. However, the adduct levels remained high in the bladder. Thus, the critical activation mechanism for a given compound may vary among different tissues.

## Effects of genetic manipulation of SULT1A1 on DNA adduct formation by genotoxicants in mouse kidney

**Figure 3:**
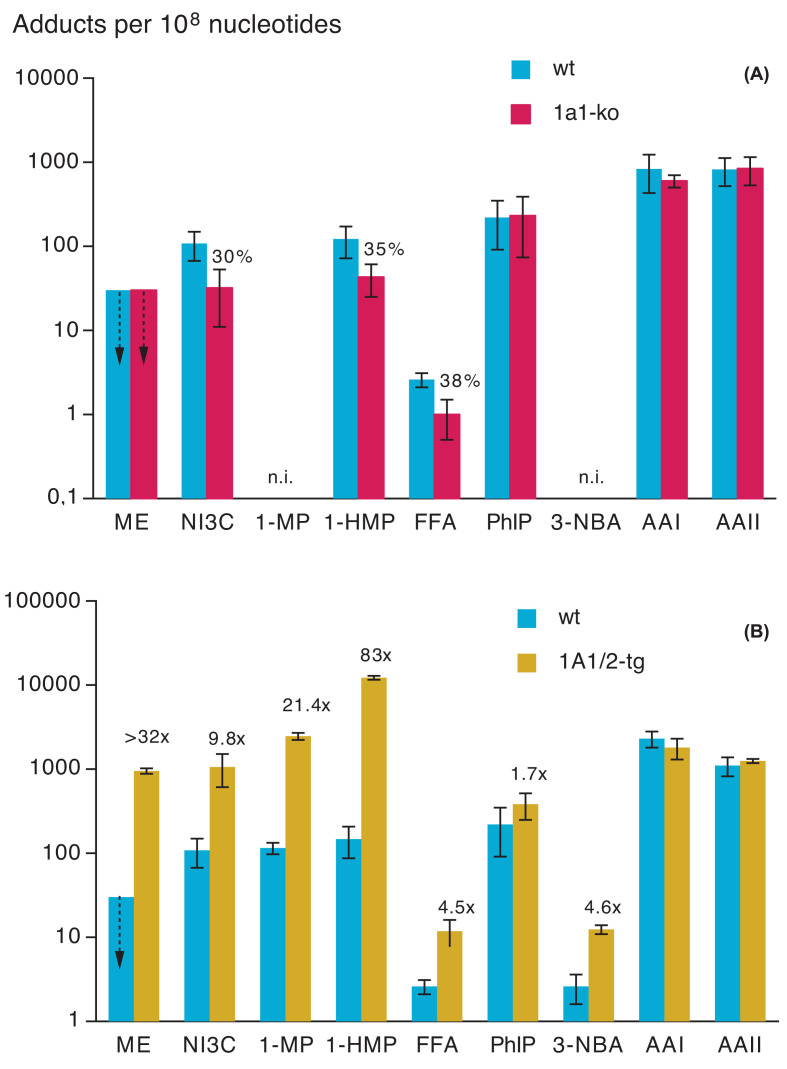
Sult1a1-knockout (upper panel) or transgenic human SULT1A1/2 (lower panel) effects on the formation of DNA adducts in the kidneys of male mice See legend to Figure 1 for all details.

The kidney deserves special attention among extrahepatic tissues, since numerous sulpho conjugates and other metabolites of xenobiotics are renally excreted. However, kidney is also equipped with xenobiotic-metabolising enzymes, including SULT1A1, 1A3 and 1B1 in humans [29,72]. In kidneys of male mice, mRNAs of various Sult isoforms were detected – with particularly high levels for Sult1d1 and rather low levels for Sult1a1 [39]. The impact of Sult1a1-ko on the formation of renal DNA adducts was investigated with seven compounds (Figure 3A). As in all other tissues, Sult1a1-ko did not affect the renal adduct formation by PhIP and aristolochic acids I and II. With methyleugenol, renal DNA adduct levels were < LOD in wt as well as Sult1a1-ko mice. Sult1a1-ko clearly reduced renal adduct formation by the remaining three compounds (to 30-35% of the wt level, Figure 3A), but less strongly than in the liver (to 2.2 to <18% of the wt level, Figure 1A). Additional study is needed to determine whether the sulpho conjugates involved were formed within the target tissue (kidney) or elsewhere (*see later section*).

tg SULT1A1/2 enhanced renal DNA adduction by seven (out of nine) genotoxicants tested (Figure 3B). For 3-nitrobenzanthrone, this enhancement was 4.6-fold in kidney, but changes were not seen in the liver (Figure 1B), speaking in favour of local activation. With five other compounds (PhIP being the exception), the SULT1A1/2-induced increase in adduct formation was stronger in the kidney than in the liver.

## Effects of Sult1d1-ko on DNA adduct formation

The SULT1D subfamily is present in humans only as a pseudogene [42]. Mouse Sult1a1 and 1d1 together satisfy many of the activities of the combined human SULT1A enzymes. The effect of Sult1d1-ko on formation of DNA adducts was investigated with six compounds. As with Sult1a1-ko, it did not affect adduct formation by the aristolochic acids in any tissue (apart from a minor increase in aristolochic acid I-induced adduction in liver) [4]. Unlike Sult1a1-ko, it generally reduced DNA adduction by PhIP in tissues with Sult1d1 expression (liver, gut, kidney) by 28–67%, but not in lung, which lacks this enzyme. Likewise, Sult1d1-ko affected adduct formation by 1-HMP in the liver, colon, and kidney, but not in the lung – although to a lesser extent than Sult1a1-ko [45]. A similar situation was encountered with the hepatic DNA adducts induced by 4-aminobiphenyl. While single ko of Sult1a1 was sufficient to diminish hepatic adduction to 1.9–4.7% of the wt level (depending on sex and treatment scheme), additional Sult1d1-ko led to a further significant decrease under all experimental conditions tested. DNA adduct formation by furfuryl alcohol was ablated by Sult1d1-ko and Sult1a1-ko in a tissue-dependent manner [7]. Thereby, Sult1d1-ko had a stronger impact than Sult1a1-ko in the small intestine.

## Effects of SULT1A1 status on other toxicological endpoints *in vivo*

Protein adducts can be determined not only in solid tissues, but also in blood proteins, and therefore represent convenient biomarkers of exposure and bioactivation in human studies [73]. Protein adducts were investigated in parallel to DNA adducts for two compounds administered to mice with differing SULT status, namely methyleugenol [74] and furfuryl alcohol [75]. With methyleugenol, hepatic protein adducts (formed with tryptophan residues) correlated with DNA adducts. Serum albumin adducts correlated with liver protein and DNA adducts. In contrast, haemoglobin adduct were low and were unaffected by the SULT status, consistent with their formation by a SULT1A-independent mechanism. The serum albumin adducts may have been formed in the liver, where this protein is synthesised. The result for furfuryl alcohol was different. In this case, adducts with the terminal valine residue of haemoglobin were determined. There was an excellent correlation between adduct levels in haemoglobin and hepatic DNA (*r*^2^ = 0.97), suggesting that 2-sulphooxymethylfuran of hepatic origin is sufficiently stable to enter circulation and penetrate the cellular membrane of erythrocytes.

Like its congener furfuryl alcohol, HMF is a food-processing contaminant that can be activated to a mutagen by SULTs expressed in recombinant bacterial and mammalian target cells [8,76]. The resulting sulpho conjugates, 5-sulphooxymethylfurfural (SMF) and 2-sulphooxymethylfuran differ greatly in their stability (*t*_1/2_ = 1.9 h and 22 s, respectively, in aqueous media at 37 °C) due to deactivation by the methoxy group present in HMF [8]. SMF could be detected in mice treated with HMF, albeit only at very low levels [77]. The renal organic anion transporters OAT1 and OAT3 mediated the cellular uptake of SMF in recombinant cell lines [78]. In fact, direct intraperitoneal (i.p.) application of SMF led to massive tubular necrosis in mice [79]. However, such histopathological effects were not seen in mice treated with HMF, not even in SULT1A1/2-tg mice, suggesting insufficient conversion to SMF [79]. However, HMF did induce DNA damage, as detected with the alkaline single-cell gel electrophoresis (comet) assay, in the kidneys of homozygous SULT1A1/2-tg mice, but not in the kidneys of wt mice and in neither the liver nor the small intestine of either mouse strain [80]. In the same study, a unique positive result was obtained with PhIP, but with liver of SULT1A1/2-tg mice as the target tissue.

1-Sulphooxymethylpyrene (1-SMP) was detected in blood plasma of rats and mice treated with 1-methylpyrene [6] or 1-HMP [45,81]. Like SMF, 1-SMP is a substrate for the renal organic anion transporters OAT1 and OAT3 [82]. Co-administration of probenecid, a typical inhibitor of OAT1 and OAT3 (and other transmembrane transporters), led to drastic increases in serum levels of 1-SMP (23-fold) and strongly altered the tissue distribution of the DNA adducts [strong increases in liver (12-fold) and lung (4-fold); slight, statistically not significant decrease in kidney (by 15%)] [81]. We conclude that 1-SMP belongs to a group of reactive sulpho conjugates that are actively transported via the circulation from the site of formation to other target tissues.

Indoxyl sulphate is a uremic toxicant suspected to be involved in the progression of renal fibrosis [46]. This sulpho conjugate is chemically stable and has no alkylating properties, unlike the sulpho conjugates considered in the preceding sections. It is formed from indole, a product of intestinal bacteria, via 3-hydroxylation catalysed by cytochromes P450 (CYPs) and sulphation catalysed by SULT1A1. Hou et al. [46] investigated the toxico-pathological role of indoxyl sulphate in renal fibrosis in Sult1a1-ko mice. Kidney damage was initiated by unilateral ureteral obstruction. This treatment led to renal accumulation of indoxyl sulphate and to inflammation and fibrosis in wt mice; all of these parameters were alleviated in Sult1a1-ko mice. In another study, Sult1a1-ko attenuated the acute kidney toxicity induced by cisplatin [47]. These findings corroborate the hypothesis that endogenous indoxyl sulphate can be nephrotoxic.

## Kinetics and extent of SULT-mediated toxification *in vivo*, competing reactions

Conjugation processes are typically much faster than CYP-mediated oxidative biotransformations. This is illustrated, *e.g*., by the time-courses determined for various SULT-mediated toxification reactions. High levels of DNA adducts were detected in mouse liver (and other tissues) already at the first time point studied, 10 min after i.p. administration of furfuryl alcohol; then, the adduct levels remained approximately constant over the following 24-h period [7]. In another study [45], wt and SULT1A1/2-tg mice were treated with 1-HMP (i.p.): DNA adduct levels in the liver (and several other tissues), as well as plasma 1-SMP, peaked within 7.5–15 min; renal DNA adducts levels reached a peak after 30 min. SMF is another reactive sulpho conjugate that could be detected in circulating blood after administration of a pro-genotoxicant. Its maximal plasma level was observed at the first sampling time, 2.5 min after intravenous administration of HMF to mice, followed by a rapid decline (by a factor of nearly 100 within 30 min) [77].

tg SULT1A1/2 strongly enhanced hepatic DNA adduction, even in the presence of Sult1a1 (*e.g*. 13.4-fold with 1-HMP, and 8.9-fold with 1’-hydroxymethleugenol), implying that only small fractions of the proximate genotoxicant (≤7.5 and 11%, respectively, in the examples) were sulpho conjugated by the endogenous Sult enzymes. Efficient competing biotransformation reactions must exist. In the case of primary alcohols (Scheme S1), oxidation (catalysed by alcohol dehydrogenases, ADHs, and subsequently by aldehyde dehydrogenases, ALDHs) was identified as an efficient competing reaction [83,84]. In fact co-administration of 4-methylpyrazole (inhibitor of ADHs), disulfiram (inhibitor of ALDHs) or ethanol (competing substrate of ADHs) strongly enhanced 1-HMP-induced formation of DNA adducts in rat liver (by factors of 27, 3.8, and 15, respectively) [85]. Analogous effects were observed in mice co-treated with furfuryl alcohol and 4-methylpyrazole or ethanol [86]; mice tg for SULT1A1/2 showed higher starting levels of DNA adducts than did wt mice, but the relative increases in the adduct levels in co-treated animals were similar – *e.g*., 4-methylpyrazole enhanced adduct formation by furfuryl alcohol in male liver 2.7- and 3.9-fold in wt and SULT1A1/2^+/−^-Sult1a1^−/−^-Sult1d1^−/−^ mice, respectively. This observation implies that even the high SULT1A1/2 levels were unable to out-compete other metabolic pathways.

Most SULT substrates are also metabolised by another class of conjugation enzymes, the UDP-glucuronosyltransferases (UGTs) (Scheme S1). In contrast with the SULTs, UGTs are rarely involved in toxification reactions. Therefore, the competition of UGTs for SULT substrates may reduce SULT-mediated toxifications. For example, 1’-hydroxymethyleugenol, the proximate genotoxicant of methyleugenol, is efficiently glucuronidated in the presence of rat liver microsomes [87]. On the one hand, extensive sequestration of the proximate genotoxicant (by ADHs, UGTs or other enzymes) will reduce adverse effects; nevertheless, sufficient toxification occurred with various SULT-dependent genotoxicants to induce tumorigenesis in animal models. On the other hand, high SULT activity or weakening of sequestration reactions (*e.g*., by ethanol for compounds toxified via primary benzylic or allylic alcohols) may have particular strong impacts on individual risk.

## Copy number variations (CNV) of *SULT* genes in humans

CNVs have been detected for two human *SULT* genes, *SULT1A1* [88–90] and *SULT2A1* [91]. One to three *SULT2A1* gene copies were observed among 30 Swedish men studied; subjects with two or three gene copies excreted higher levels of DHEA sulphate and androsterone sulphates in their urine than did individuals with only one gene copy [91]. CNVs of *SULT1A1* have been studied in much larger populations; the gene copy numbers reported were 0 (for a single subject [90]), 1, 2, 3, 4, or ≥ 5. Thus, the presence of a functional *SULT1A1* gene is not essential for humans, as in mice. The CNVs were associated with the levels of SULT1A1 mRNA, protein, and enzyme activity in human liver specimens [88,89].

The methyleugenol DNA adducts that have been studied in mice with varying SULT1A status were also detected in all 121 human liver specimens analysed by Tremmel *et al*. [89]. The adduct levels, varying 122-fold among the individuals, were highly significantly correlated with SULT1A1 mRNA and protein levels as well as with gene copy numbers. These methyleugenol DNA adducts were also detected in all ten human lung specimens examined [92]; in addition DNA adducts of furfuryl alcohol were detected in the lung samples [92]. A definitive study of the possible correlation of these pulmonary DNA adducts with SULT1A1 status (*e.g*. CNV) would require larger numbers of samples. Nevertheless, these findings corroborate that SULT-dependent activation occurs in humans and may lead to DNA damage in liver as well as extrahepatic tissues.

## Conclusions

Studies in SULT-ko and -tg mouse strains have demonstrated that SULTs are pivotal for the bioactivation of several genotoxicants in that species. Although mice have 21 functional *Sult* genes, a single isoform may overwhelmingly dominate the activation of a substance in relevant tissues, as has been explicitly demonstrated for Sult1a1. We consider the most relevant tissues to be those that are major targets for tumorigenicity and those with particularly high DNA adduct levels. The effects of genetic manipulation of SULT status were primarily seen in tissues with high expression of the corresponding enzyme – differing but overlapping for Sult1a1, Sult1d1, and SULT1A1/2. In addition, some reactive (or otherwise toxic) sulpho conjugates may be distributed in the organism via the circulation, primarily to the kidneys. This was seen most clearly for the cases of 1-SMP, SMF, and indoxyl sulphate, whose presence was directly detected in blood. Moreover, all of these metabolites were found to be substrates of OAT1 and OAT3 [78,82,93], which mediate the uptake of various organic anions from the basolateral (blood) site into proximal tubule cells, resulting in particularly strong DNA adduction and/or other adverse effects at this site. Further evidence for transfer of reactive sulpho conjugates was obtained with furfuryl alcohol: it formed haemoglobin adducts, whose levels were highly correlated with levels of hepatic DNA adducts [75]. Kidney is the target tissue for furfuryl alcohol-induced tumorigenesis in mice [70]. However, it cannot yet be concluded to what extent the renal DNA adducts (and tumours) were a consequence of local bioactivation versus reactive metabolites formed elsewhere and then transferred to the kidneys.

tg SULT1A1/2 strongly enhanced the DNA adduction even in the presence of Sult1a1, indicating that only a limited fraction of the proximate genotoxicant was sulpho conjugated by the endogenous Sult enzymes, and that sequestration via alternative pathways (such as oxidation by ADH, or conjugation with glucuronic acid by UGT, *see* Scheme S1) may substantially attenuate the activation. Despite this possible attenuation, various SULT-dependent compounds are carcinogenic in animal studies. Thus, high SULT activity or impairment of sequestering reactions may reinforce the individual risk of adverse effects of such compounds in humans.

*SULT1A1* CNV is common in humans (even – although very rarely – *n* = 0, *i.e.*, the equivalent of a homozygous ko). The CNV strongly affects SULT1A1 expression. Methyleugenol DNA adducts detected in human liver specimens were correlated with SULT1A1 expression and gene copy numbers.

tg SULT1A1/2 enhanced DNA adduction not only in liver, but also in many extrahepatic tissues, in line with its wide expression. Thus, humans may have more target tissues for SULT1A1/Sult1a1-dependent genotoxicants than do (wt) mice. Indeed, DNA adducts of SULT-dependent genotoxicants have been detected in extrahepatic human tissue samples, using highly specific LC-MS/MS methods. They include adducts of furfuryl alcohol and methyleugenol in the lung [92] and adducts of PhIP in the prostate [94].

## Summary

- It is generally accepted in toxicology that numerous carcinogens, mutagens and other toxicants are not active as such, but require metabolic activation, often to electrophilic intermediates. Most toxicologists primarily associate activation reactions with CYPs, whereas conjugation reactions are seen as detoxification processes. However, in this review we provide direct or circumstantial evidence for a critical role of SULTs in the activation of hundreds of chemicals in *in vitro* models.
- Using mouse lines with Sult-ko and/or human SULT transgenes, we demonstrate for selected compounds – most of them established carcinogens in mouse liver and/or kidney – a dominant role of SULTs in the bioactivation *in vivo*, confirming *in vitro* findings.
- Although mice have 21 functional *Sult* genes, knockout of a single isoform, Sult1a1, was sufficient to reduce adverse effects (DNA adduct formation) of several test compounds by >95%.
- SULT-dependent DNA adducts formation was often restricted to tissues with fair expression of the corresponding SULT isoform (*e.g*. liver and large intestine for mouse Sult1a1; additionally, small intestine, lung, and kidney for human SULT1A1/2). These local effects of reactive metabolites at the site of their formation can be explained by the fact that sulpho conjugates are charged molecules that do not penetrate cell membranes passively.
- However, many sulpho conjugates are substrates for transmembrane transporter proteins. Thus, some toxic sulpho conjugates were sufficiently stable to be transported via the circulation to the kidneys where they formed DNA adducts or produced other adverse effects.
- Many standard *in vitro* models ignore SULT-mediated activation. It is important to include SULT-proficient models, at least when testing compounds with structural alerts for SULT-mediated activation.

## Abbreviations

1-HMP: 1-hydroxymethylpyrene
1-SMP: 1-sulphooxymethylpyrene
ADH: alcohol dehydrogenase
AHBAs: compounds sharing a core structure of 4-amino 2-halogenated benzyl alcohol
ALDH: aldehyde dehydrogenase
CNV: gene copy number variation
CYP: cytochrome P450
HMF: 5-hydroxymethylfurfural
i.p.: intraperitoneal
ko: knockout
LC-MS/MS: liquid chromatography coupled with tandem mass spectrometry
LOD: limit of detection
NAT: N,O-acetyltransferases
NI3C: *N*-methoxy-indole-3-carbinol
OAT: organic anion transporter
p.o.: *per os* (oral)
PAPS: 3'-phosphoadenosine-5'-phosphosulphate
PhIP: 2-amino-1-methyl-6-phenylimidazo[4,5-*b*]pyridine
RAL: relative adduct level detected using the ^32^P-postlabelling analysis
SMF: 5-sulphooxymethylfurfural
SULT: cytosolic sulphotransferase (generic or human forms)
Sult: cytosolic sulphotransferase (mouse forms)
tg: transgenic
UGT: UDP-glucuronosyltransferases
YC-1: lificiguat
wt: wild-type
